# PAK1-mediated phosphorylation of ARGLU1 promotes tumorigenic potential of breast cancer

**DOI:** 10.1016/j.jbc.2026.113491

**Published:** 2026-08-25

**Authors:** Srikanth Swamy Swaroop B, Prarthana Gopinath, Rahul Kanumuri, Abirami Seetharaman, Roshni Saravanan, Sandhya Sundaram, Suresh Kumar Rayala, Ganesh Venkatraman

**Affiliations:** 1 Department of Biotechnology, Indian Institute of Technology Madras (IITM), Chennai, India; 2 Department of Pathology, Sri Ramachandra Medical College, Sri Ramachandra Institute of Higher Education and Research, Chennai, Tamil Nadu, India; 3 Department of Bio-Medical Sciences, School of Bio Sciences & Technology, Vellore Institute of Technology, Vellore, Tamil Nadu, India

**Keywords:** PAK1, ARGLU1, phosphorylation, breast cancer, kinase interacting substrate strategy

## Abstract

P21-activated kinase1 (PAK1) is an oncogenic kinase that is often hyper-activated or overexpressed in multiple cancers including breast cancer (BC). By phosphorylating a diverse range of substrates, PAK1 promotes tumor progression through multiple signaling pathways. Herein, we report arginine and glutamate rich-protein 1 (ARGLU1), a protein that is overexpressed in BC, as a novel substrate of PAK1. Through mass spectrometry, PhosTag, and metabolic labeling of cells with P^32^-orthophosphoric acid, we identified and confirmed that PAK1 phosphorylated ARGLU1 at Ser 77. Furthermore, to investigate the impact of this phosphorylation, we created BC cells with ARGLU1 Ser 77 to Ala mutation that resulted in lower E-box promoter activity, lower expression of MCM genes, and higher G2/M fraction and this was rescued by the phosphomimic mutant (Ser 77 to Asp). Furthermore, BC cells that stably expressed the phosphor inactive form of ARGLU1 displayed lower proliferation rates along with diminished ability to form colonies, migrate, invade, and form spheroids indicating the significance of the phosphorylation. Furthermore, ARGLU1 was found to be overexpressed in BC samples and clinical data from publicly available databases also implied that pARGLU1 S77 was not only elevated in BC, but also was associated with advanced stages and poor overall survival. The present study provides evidence that ARGLU1 is a novel substrate of PAK1 and its phosphorylation at S77 contributes to BC pathogenesis.

Breast cancer (BC) is the most frequent malignancy among women, with an estimated 2.3 million new cases worldwide in 2022 (1). Research in the past decades has helped to understand the highly heterogeneous nature of the disease at the molecular level and thus has pivoted the treatment towards biology-informed approach (2). Despite several advancements in the theranostics, GLOBOCAN 2022 suggests that the BC ranks fourth among all the cancer related mortalities (1). Identifying newer pathways involved in BC initiation and/or progression, which can further be targeted becomes a necessity. p21-activated kinase 1 (PAK1) is a Ser/Thr kinase, initially identified as a downstream effector of Cdc42 and Rac1 (3). It phosphorylates a multitude of substrates, thereby involving in diverse cellular processes like growth factor signaling, transcriptional regulation, cell cycle progression, and cytoskeletal remodeling (4). PAK1 overexpression or hyperactivation is reported in a variety of cancers (5). The upregulated PAK1 signaling is often due to gene amplification or dysregulated transcription or point mutations in the upstream regulators leading to hyperactivation (6). The *Pak1* gene is located in the chromosomal region 11q13, which is often found to be amplified in BC (https://aacrjournals.org/cancerres/article-abstract/52/19/5229/498015/Amplification-of-Genes-within-the-Chromosome-11q13?redirectedFrom=fulltext). Also, Wang *et al.* reported that hyperactivation of PAK1 could lead to the formation of the tumors of the mammary gland in transgenic mice model (7). Studies accumulated over the years indicate that PAK1 impacts multiple hallmarks of cancer such as promoting autonomous growth signals, evading growth suppressing signals, increasing cell survival, promoting cellular motility and metastasis (8, 9). Moreover, the role of PAK1 in promoting resistance to tamoxifen (10, 11), gemcitabine (12, 13), fulvestrant, and abemaciclib (14) is also well documented. Thus, discovering new substrates of PAK1 would help us understand the intricate molecular mechanisms by which PAK1 promotes tumorigenesis and therapy resistance.

In the current study, we report arginine and glutamate-rich protein 1 (ARGLU1) as a novel substrate of PAK1. ARGLU1 is a 33 kDa, highly disordered protein, with arginine-rich N-terminal and glutamate-rich C-terminal. It was initially reported as a transcriptional coactivator of estrogen receptor α by interacting with MED1 (15). Further study by Magomedova *et al.* showed that ARGLU1 acts as a splicing regulator and a transcriptional coactivator of glucocorticoid receptor (16). ARGLU1 was reported to inhibit gastric cancer progression through SP1 and YY1 mediated upregulation of DNA mismatch repair genes (17). More recently, ARGLU1 overexpression was found to promote proliferation and resistance to genotoxic drugs in fibrosarcoma (18). While ubiquitination-mediated degradation led to head and neck squamous cell carcinoma (HNSCC) progression, other posttranslational modifications of ARGLU1 have not been much investigated (19). In this study, we explored PAK1-mediated phosphorylation of ARGLU1 and the resultant functional and clinical implications in BC.

## Results

### ARGLU1 is a physiologically interacting novel substrate of PAK1

In an attempt to identify novel substrates of PAK1, we performed kinase-interacting substrate screening (KISS) assay in the BC cell line - MDA-MB-231. *Pak1* KO MDA-MB-231 generated previously by our group (20) was used as the experimental model system to minimize the possible phosphorylation by endogenous PAK1 (Figs. 1*A* and S1*A*). From the resulting data, a cut-off of ≥ 3 was set for the number of validated peptide spectrum matches to shortlist the phosphopeptides with high stringency. Analysis revealed 88 phosphosites belonging to 67 phosphopeptides in the (+) ATP reaction. In the control, (−) ATP reaction, we identified 36 phosphosites belonging to 19 phosphopeptides. The identified candidate substrates unique from (+) ATP reactions are summarized in Figure 1*B* and Table S1. Detection of known substrates of PAK1 such as ARGHEF6 (α-PIX), ARGHEF7 (β-PIX), and NLRP3 suggested the validity of this approach. ARGLU1 was found as one of the potential substrates of PAK1 (Fig. 1*B*).

**Figure 1:**
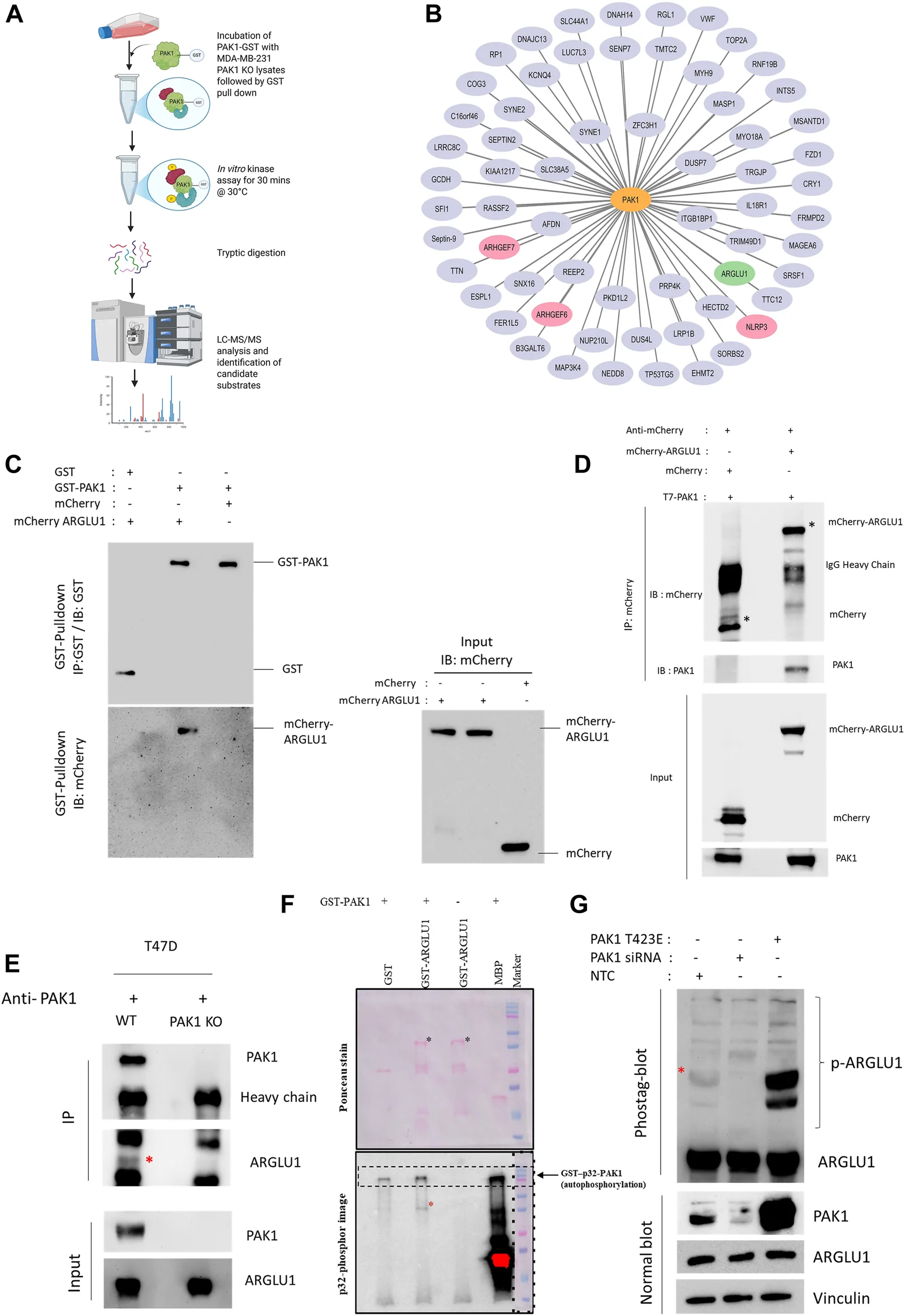
**ARGLU1 is a physiological substrate of PAK1.***A*, schematic representation of kinase-interacting substrate screening (KISS) Assay workflow. *B*, schematic representation of potential substrates of PAK1 identified by KISS approach. *Red circles* indicate known PAK1 substrates, and ARGLU1 is highlighted in *green*. *C*, immunoblot of GST-pull down assay. *D*, immunoblot of coimmunoprecipitation of PAK1 with anti-mCherry pulldown in HEK293T cells cotransfected with T7-PAK1 and mCherry or mCherry-ARGLU1. *E*, immunoblot of Coimmunoprecipitation of endogenous ARGLU1 with anti-PAK1 in T47D WT and T47D PAK1 KO cells. *F*, *in vitro* kinase assay with γP32 ATP. Myelin basic protein (MBP) was used as positive control and GST protein as negative control. Ponceau blot (*upper panel*) shows the presence of GST-ARGLU1 protein. Phosphor image (*lower panel*) shows phosphorylation of GST-ARGLU1 only in the presence of GST-PAK1. Autophosphorylation of GST-PAK1 also serves as an additional positive control. *G*, PhosTag blot indicating change in the phospho-ARGLU1 levels upon silencing (PAK1 siRNA) and overexpressing PAK1 (constitutively active form of PAK1 T423E). ARGLU1, arginine and glutamate-rich protein 1; PAK1, p21-activated kinase 1.

To recapitulate KISS assay and validate interaction of PAK1 with ARGLU1, we performed GST pull down assay. Briefly, we incubated mCherry-ARGLU1 with recombinant GST or GST-PAK1 proteins. The GST proteins were pulled down using GST beads. mCherry-ARGLU1 coeluted with GST-PAK1 upon GST pull down, indicating direct physical interaction between PAK1 and ARGLU1 (Fig. 1*C*). To assess the interaction *in vivo*, coimmunoprecipitation from HEK293T cells transiently overexpressing mCherry or mCherry – ARGLU1 and T7-PAK1 and was subjected to anti-mCherry pull-down. PAK1 was found to be coimmunoprecipitated only with mCherry-ARGLU1 (Fig. 1*D*). To further strengthen the evidence of *in vivo* interaction between PAK1 and ARGLU1, we performed endogenous coimmunoprecipitation with anti-PAK1 in T47D cells with PAK1 KO T47D as control. ARGLU1 was detected in the subsequent immunoblot exclusively in the WT T47D (Fig. 1*E*).

*In vitro* kinase assay set up with ϒP^32^- ATP showed radioactive phosphorylation band only in the presence of GST-PAK1 and not in the control reaction set up without the kinase (Fig. 1*F*). To substantiate this phosphorylation occurs *in vivo, PAK1* was knocked down and overexpressed in T47D cells and the lysates were run on Mn^2+^ PhosTag gel. In a PhosTag gel, phosphorylated proteoforms are retarded owing to the binding of PhosTag acrylamide to the negative phosphate moieties. We observed a stark reduction in the intensity of the phosphorylation band in samples transfected with the *PAK1* siRNA. The intensity of the band increased in the samples transfected with constitutively active PAK1T423E, when compared to the siRNA control as well as the PAK1 siRNA transfected samples. This confirmed the PAK1-specific phosphorylation of ARGLU1 occurs in the cellular context (Fig. 1*G*). Taken together, these experiments confirmed that PAK1 interacts with and phosphorylates ARGLU1 under physiological conditions.

### PAK1 phosphorylates ARGLU1 at Ser 77

By KISS assay, we derived that PAK1 phosphorylated ARGLU1 at Ser77 (Fig. 2*A*). The sequence, RASSPPD was also found to contain the PAK1 consensus sequence, R/KXXS/T. Multiple sequence alignment showed that the RASSPPD motif, which includes the Ser77 position, is highly conserved across species (Fig. 2*B*) indicating its functional significance. However, mass spectrometry–based proteomics data require rigorous validation to ensure the accuracy, specificity, and reproducibility, due to the inherent caveats associated with high-throughput analytical workflows. Therefore, to validate the site of PAK1 phosphorylation, we performed three independent experiments. Firstly, we designed and commercially procured two peptides- WT (DRERASSPPDRIDIFGRTVS) and S77A (DRERASAPPDRIDIFGRTVS) and set up an *in-vitro* kinase assay with or without GST-PAK1. Matrix-assisted laser desorption ionization-mass spectrometry analysis of the peptides showed the phosphorylation only in WT in the presence of GST-PAK1. Phosphorylation of the serine residue adds +79.9663 Da to the protein/peptide. WT with a prominent parental peak at 2274.59 Da showed a peak at 2354.54 Da (+79.95 Da) denoting the phosphorylation, and it was absent in the S77A (Fig. 2, *C* and *D*).

**Figure 2:**
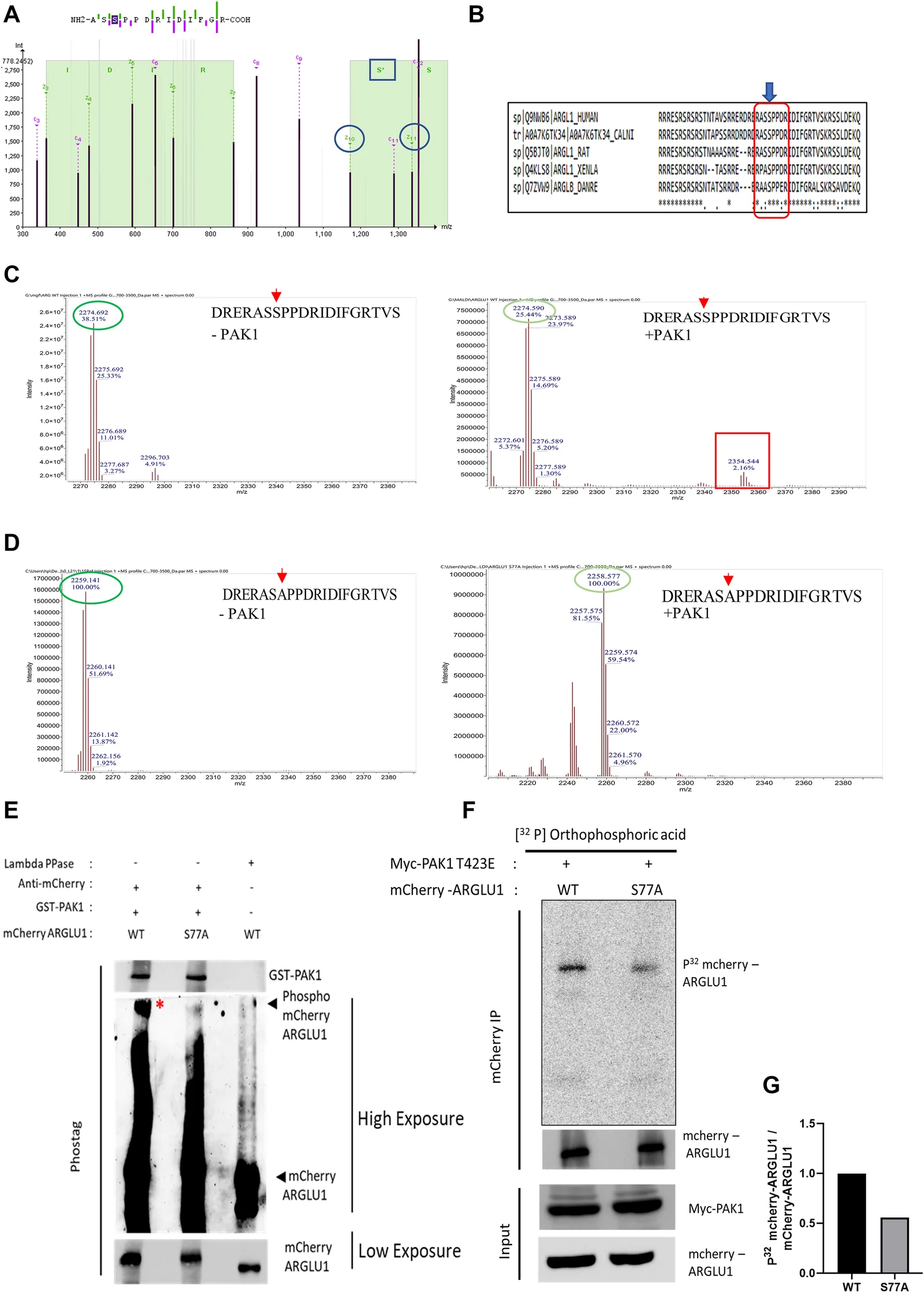
**PAK1 phosphorylates ARGLU1 at Serine 77.***A*, representative MS/MS spectra from KISS assay indicating phosphorylation of ARGLU1 at Ser77. *B*, multiple sequence alignment indicating that the Ser77 site is highly conserved across species. *C*, MALDI-TOF MS spectra of the ARGLU1 WT peptides. The *left panel* indicates spectra of the reaction incubated without PAK1 and the *right panel* indicates spectra of the reaction incubated with PAK1. *D*, MALDI-TOF MS spectra of the ARGLU1 S77A peptides. The *left panel* indicates spectra of the reaction incubated without PAK1 and the *right panel* indicates spectra of the reaction incubated with PAK1. *E*, *in vitro* kinase assay with GST-PAK1 and mCherry pulldown from HEK293T cells, followed by PhosTag immunoblot indicating the absence of phosphorylation band in mCherry-ARGLU1 S77A. λ-Phosphatase treated lysate was used as control for unphosphorylated ARGLU1. *F*, *in vivo* metabolic labeling with P32 orthosphosphoric acid showing differential intensity between mCherry-ARGLU1 WT and mCherry-ARGLU1 S77A cotransfected with constitutively active PAK1 T423E. *G*, graphical representation of the intensity of the P32-labeled mCherry-ARGLU1 using densitometric analysis, normalized to the total mCherry-ARGLU1 pulldown. ARGLU1, arginine and glutamate-rich protein 1; MALDI-TOF MS, matrix-assisted laser desorption/ionization time-of-flight mass spectrometry; PAK1, p21-activated kinase 1.

Secondly, we generated phospho-deficient mutant of ARGLU1 by mutating the serine 77 to alanine (called ARGLU1 S77A henceforth). We transfected the mCherry-ARGLU1 WT or mCherry-ARGLU1 S77A plasmids into HEK293T cells and immunoprecipitated mCherry. On-bead *in-vitro* kinase assay was set up with GST-PAK1 for the immunoprecipitated samples followed by PhosTag gel analysis. The presence of a prominent phosphorylation band uniquely in mCherry-ARGLU1 WT immunoprecipitate showed that PAK1 phosphorylated ARGLU1 at Ser77. λ-Phosphatase treated lysate was used as control for unphosphorylated mCherry-ARGLU1 (Fig. 2*E*). Thirdly, mCherry-ARGLU1 WT or mCherry-ARGLU1 S77A plasmids were cotransfected with constitutively active PAK1 T423E into HEK293T cells and metabolically labeled with P^32^ orthophosphoric acid. Pull-down of these cell lysates with anti-mCherry showed an intense band in mCherry-ARGLU1 WT and a faint band (approximately 2-fold reduction) in the mCherry-ARGLU1 S77A in phosphor image (Fig. 2, *F* and *G*). Taken together, it can be inferred that ARGLU1 is phosphorylated by PAK1 at Ser 77, with possible functional and clinical relevance.

### PAK1 influences subcellular distribution of ARGLU1

Earlier studies have indicated that ARGLU1 is predominantly localized in the nucleus (15). Driven by curiosity, we attempted to investigate whether PAK1-mediated phosphorylation alters the subcellular localization of ARGLU1. We first treated the MCF-7 cells with the PAK1 inhibitor – IPA3 (20 μM) for 30 min and processed the cells for immunofluorescence staining. Laser scanning confocal microscopic analysis revealed that upon IPA-3 treatment, ARGLU1 was predominantly localized in the cytoplasm, whereas in the vehicle control it was principally localized in the nucleus (Fig. 3, *A* and *B*). To biochemically validate this finding, we knocked down *Pak1* in T47D cells and performed subcellular fractionation to separate nuclear and cytosolic compartments. Western blot analysis showed at least 4-fold reduction in the nuclear levels of ARGLU1 upon *Pak1* knockdown compared to the control (Fig. 3, *C* and *D*). As PAK1 phosphorylated ARGLU1 at Ser77, we wanted to assess whether this phosphorylation event influences nuclear localization of ARGLU1. Surprisingly, when the mCherry-ARGLU1, mCherry-ARGLU1 S77A, or mCherry-ARGLU1 S77D plasmid was transfected into HEK293T cells, nuclear exclusion of ARGLU1 S77A was not observed as expected. However, there was a differential distribution within the nucleus among the three groups. All three groups formed puncta in the nuclei, as commonly observed among the transcriptional coactivators. The signal intensities from the puncta with reference to the total signal intensity were quantified among the three groups, and revealed significant differences. It was observed that mCherry-ARGLU1 S77A had the highest percentage of signal from puncta, followed by mCherry-ARGLU1 WT (Fig. 3, *E* and *F*). Interestingly, mCherry-ARGLU1 S77D was mostly well-dispersed within the nucleus forming the lowest percentage of nuclear condensates. These findings conclude that PAK1-mediated ARGLU1 phosphorylation at Ser77 is essential for the optimal distribution within the nucleus and hence the functions of ARGLU1.

**Figure 3:**
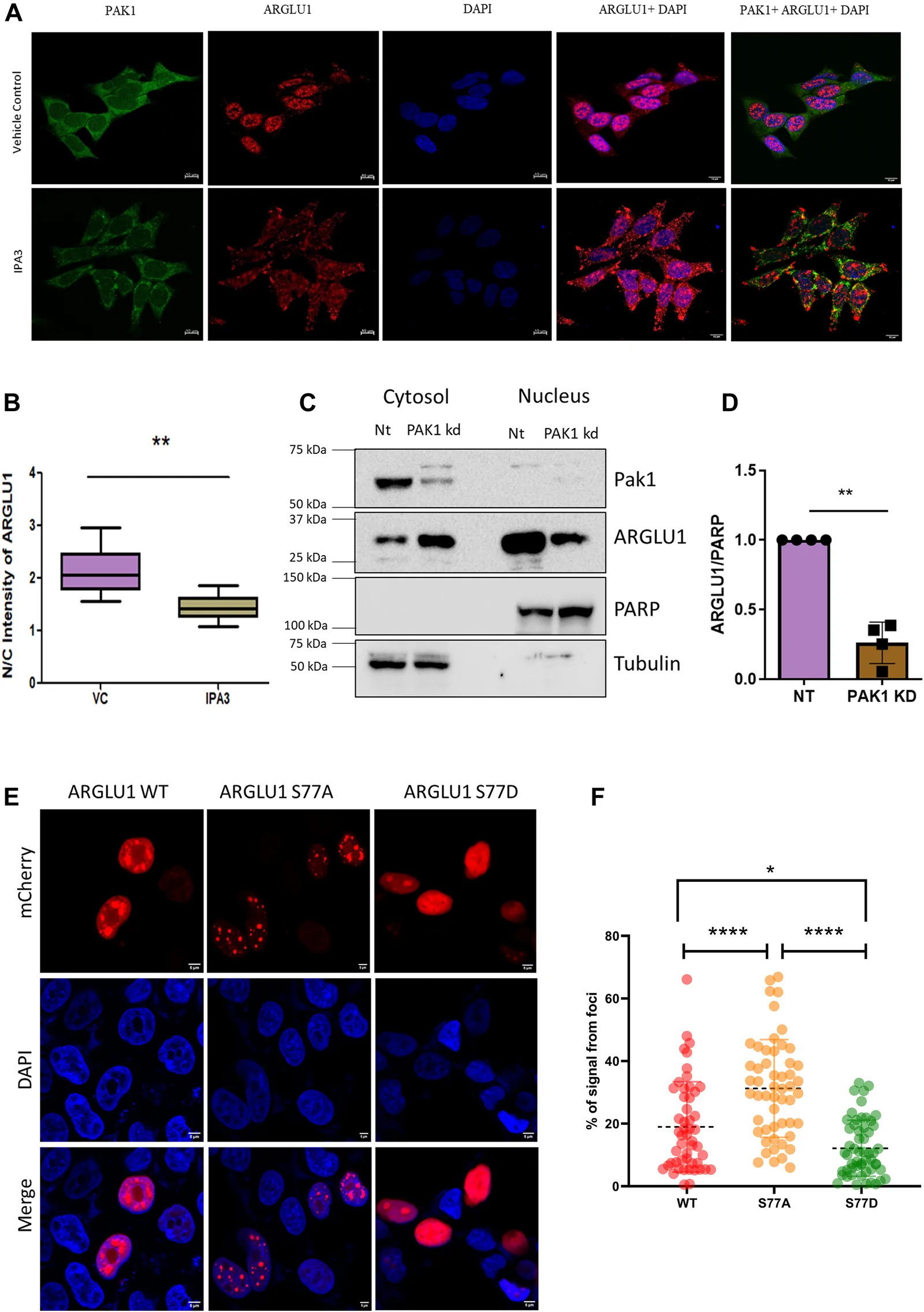
**PAK1 influences subcellular localization of ARGLU1.***A*, representative immunofluorescence images showing altered localization of ARGLU1 (*red*) upon pharmacological inhibition of PAK1 (*green*) with IPA3 in MCF-7 cells. *B*, graphical representation of ARGLU1 localization between nucleus and cytoplasm. Staining intensity of ARGLU1 in control *versus* IPA3-treated cells was plotted (n = 3 fields × 3 replicates), represented as mean ± SD and ∗∗*p* < 0.01. *C*, representative immunoblot showing declined levels of ARGLU1 in nucleus upon silencing PAK1. *D*, graphical representation of ARGLU1 expression in nucleus, normalized to PARP expression (N = 4). Bars represent mean ± SD and ∗∗*p* < 0.01. *E*, representative confocal images showing differential distribution of mCherry-ARGLU1 between WT, S77A, and S77D in the nucleus. *F*, graphical representation of the signal quantified from the puncta with respect to total signal intensity in a minimum of 50 cells. ∗*p* < 0.05, ∗∗∗∗*p* < 0.0001. ARGLU1, arginine and glutamate-rich protein 1; PAK1, p21-activated kinase 1.

### PAK1-ARGLU1 axis influences cMyc responsive transcriptional program

ARGLU1 being a known transcriptional regulator, we were captivated to explore the role of phosphorylation of ARGLU1 at Ser77 in regulating the overall gene expression landscape in clinical samples. Therefore, we analyzed the mRNA levels of the genes that correlated with the pS77- ARGLU1 levels in BC tissues from publicly available datasets – CPTAC-BRCA and TCGA–BRCA (Tables S2 and S3 respectively). To delineate the effect of ARGLU1 phosphorylation, Gene Set Enrichment Analysis was performed for total and pS77-ARGLU1 in both the cohorts (Tables S4 and S5 respectively). Normalized enrichment scores thus obtained were compared between total ARGLU1 and pS77-ARGLU1 (Fig. 4*A*). In both the cohorts, the normalized enrichment scores of E2F targets, G2/M checkpoint, MYC targets variant 1 showed positive correlation with total ARGLU1 with a false discovery rate < 0.05. Importantly, MYC targets variant 1 showed stronger association with pS77-ARGLU1 levels (Fig. 4, *B* and *C*). Building on these findings, we confirmed the interaction of ARGLU1 and cMyc using coimmunoprecipitation, with FLAG-ARGLU1 and HA-Myc (Fig. 4*D*). We also established colocalization of ARGLU1 with cMyc using immunofluorescence (Fig. 4*E*).

**Figure 4:**
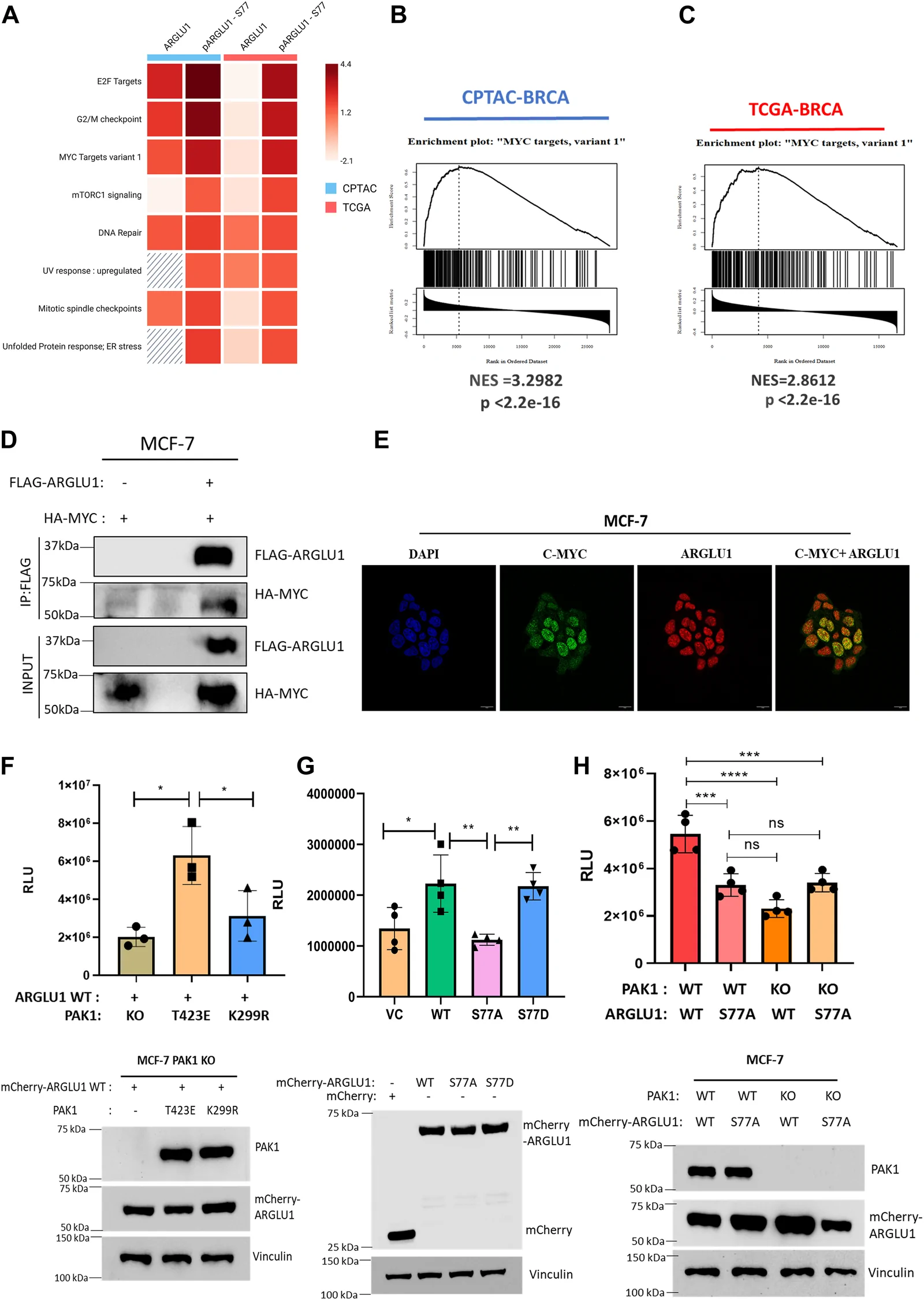
**Phosphorylation of ARGLU1 at Ser 77 affects cMyc regulated transcriptional program.***A*, heatmap of differentially regulated pathways between ARGLU1 and pS77-ARGLU1 from TCGA and CPTAC BRCA datasets. Each tile represents the normalized enrichment score. *B* and *C*, enrichment plots of “MYC targets variant 1” for the genes correlating with pS77-ARGLU1 in CPTAC and TCGA BRCA cohorts. *D*, immunoblot indicating coimmunoprecipitation of HA-Myc with FLAG-ARGLU1 pulldown in MCF-7 cells cotransfected with HA-Myc and FLAG-ARGLU1. *E*, immunofluorescence microscopy indicating colocalization of c-Myc with ARGLU1 in MCF-7 cells. The scale bar represents 20 μm. *F*, *upper panel*: graphical representation of luciferase activity in MCF-7 PAK1 KO cells cotransfected with 5 X E-box promoter luciferase construct, mCherry-ARGLU1 WT, and PAK1T423E or PAK1K299R plasmids. mCherry-ARGLU1 WT alone transfected cells served as baseline, and transfections were normalized using β-Galactosidase activity. *lower panel*: representative immunoblot indicating transfections in the lysates used for the luciferase assay. *G*, *upper panel*: graphical representation of luciferase activity in MCF-7 cells stably expressing mCherry (VC), mCherry-ARGLU1 WT, mCherry-ARGLU1 S77A, and mCherry-ARGLU1 S77D transfected with 5 X E-box promoter luciferase constructs. Transfections were normalized using β-Galactosidase activity. *Lower panel*: representative immunoblot probed with anti-mCherry in the lysates used for the Luciferase assay. *H*, *upper panel*: graphical representation of luciferase activity in MCF-7 WT and PAK1 KO cells cotransfected with 5 X E-box luciferase constructs and mCherry-ARGLU1 WT or S77A constructs. Transfections were normalized using β-Galactosidase activity. *Lower panel*: representative immunoblot confirming PAK1 KO and transfections in the lysates used for the luciferase assay. Bars represent mean ± SD. Statistical analysis performed was one-way ANOVA with Tukey’s posttest for multiple comparisons. ∗*p* < 0.05, ∗∗*p* < 0.01, ∗∗∗*p* < 0.001, and ∗∗∗∗*p* < 0.0001. ARGLU1, arginine and glutamate-rich protein 1; CPTAC, Clinincal Proteomic Tumor Analysis Consortium; TCGA, The Cancer Genome Atlas.

To evaluate the role of PAK1-ARGLU1 axis in regulating cMyc responsive transcription, we used E-box luciferase promoter constructs. Firstly, to assess the role of PAK1 on the E-box activity, we cotransfected ARGLU1 WT with constitutively active PAK1 (T423E) or kinase-dead PAK1 (K299R) in PAK1 KO MCF-7 cells. In the presence of PAK1 T423E, approximately 3-fold increase in the relative light units (RLUs) was observed compared to control (only ARGLU1 WT transfected in PAK1 KO MCF-7 cells), the RLU of cells transfected with PAK1 K299R showed no significant difference from the control group (Fig. 4*F*). To summarize, PAK1 phosphorylation plays significant role in ARGLU1-mediated E-box promoter activity. Secondly, the significance of the ARGLU1 pS77 was assessed using the luciferase assay in MCF-7 cells expressing mCherry (vector control- VC), mCherry-ARGLU1 WT (WT), mCherry-ARGLU1 S77A (S77A), and mCherry-ARGLU1 S77D (S77D). As expected, WT cells showed ∼2-fold increase in the activation of E-box promoter, when compared to VC. Phospho-null mutant S77A showed sharp decline in the RLU, comparable to VC, which was rescued in phospho-mimic S77D mutant (Figs. 4*G* and S1*B*). Hence, it can be ascertained that phosphorylation of ARGLU1 at S77 plays a vital role in the E-box promoter activity.

Finally, to explore if the alteration in the E-box activity is because of the phosphorylation of ARGLU1 at S77 by PAK1, ARGLU1 WT and S77A was transfected into both MCF-7 WT and PAK1 KO cells. ARGLU1 WT transfected MCF-7 WT cells demonstrated the maximum promoter activity among the study groups. Unsurprisingly, PAK1 KO cells showed lower RLU, with no significant difference between ARGLU1 WT and S77A. The activation of E-box in both ARGLU1 WT and S77A transfected PAK1 KO cells was analogous to that of S77A transfected MCF-7 WT cells. In the absence of PAK1, no significant difference was observed with both WT and S77A ARGLU1 (Fig. 4*H*). Taken together, we conclude that ARGLU1 binds to cMyc and plays a significant role in E-box promoter activity, and this is dependent on the PAK1 phosphorylation of ARGLU1 at S77.

To further validate the role of ARGLU1 phosphorylation in regulating cMyc responsive genes, we attempted to assess the expression levels of MCM genes. MCM family of genes contain E-box sequence in their promoter and are known to be regulated by cMyc (Fig. 5, *A* and *B*). Therefore, we evaluated the expression of MCM genes among VC, WT, S77A, and S77D-expressing cells by qRT-PCR. As expected, WT was found to express significantly higher levels of MCM gene when compared to VC. Predictably, S77A cells showed a sharp and significant decline in the gene expression, which was salvaged in the phospho-mimic S77D cells (similar to WT). Among the analyzed genes, MCM7 was the most dysregulated with almost 10-fold increase in the WT and S77D cells compared to VC and S77A (Fig. 5, *C*–*F*). Moreover, among the differentially regulated genes from the TCGA and CPTAC dataset yielded MCM genes to be more positively associated with ARGLU1 pS77 (Fig. 5, *G* and *H*). With the current evidence, we can conclude that ARGLU1 pS77 plays a significant role in the regulation of MCM gene expression.

**Figure 5:**
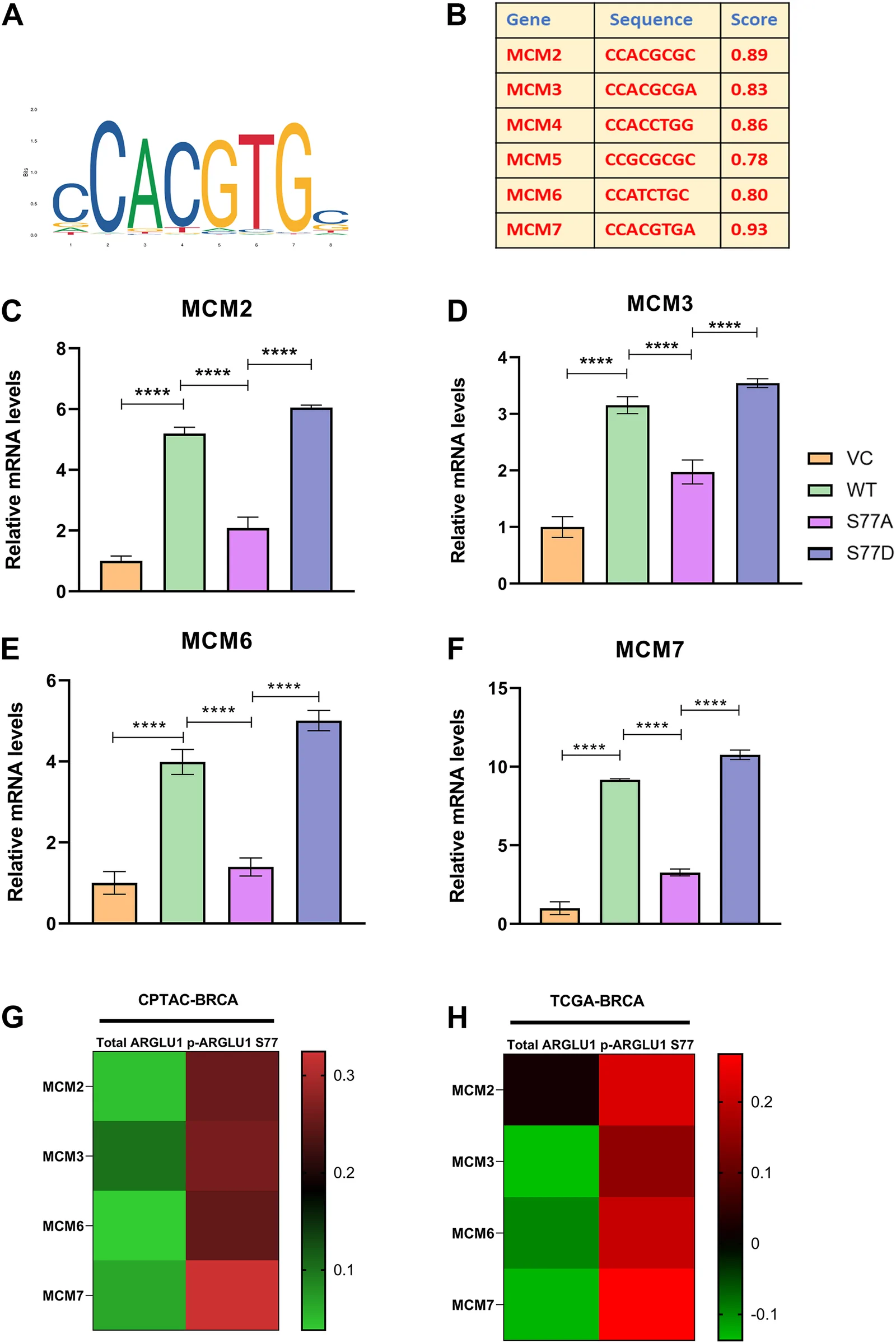
**Phosphorylation of ARGLU1 at Ser77 upregulates cMyc responsive genes.***A*, schematic representation of canonical E-box sequence acquired from JASPAR v.MA0147.4, known to be responsive to cMyc. *B*, tabular representation of E-box sequence found in the promoter region (−500 to +100 bp, relative to transcription start site) of MCM genes. A score ≥ 0.8 is considered more favored. *C*, *D*, *E*, and *F*, graphical representation of MCM2, 3, 6, and 7 mRNA levels normalized to β-Actin using qRT-PCR in MCF-7 cells expressing mCherry (VC), mCherry-ARGLU1 WT, mCherry-ARGLU1 S77A, and mCherry-ARGLU1 S77D. Bars represent mean ± SD and n = 3. Statistical analysis performed was one-way ANOVA with Tukey’s posttest for multiple comparisons. ∗*p* < 0.05, ∗∗*p* < 0.01, ∗∗∗*p* < 0.001, and ∗∗∗∗*p* < 0.0001. *G* and *H*, heatmap showing co rrelation of MCM genes with total and pS77- ARGLU1 in CPTAC and TCGA BRCA cohorts. Each tile represents Spearman correlation coefficient. ARGLU1, arginine and glutamate-rich protein 1; CPTAC, Clinincal Proteomic Tumor Analysis Consortium; MCM, minichromosome maintenance; TCGA, The Cancer Genome Atlas.

### Phosphorylation of ARGLU1 at Ser77 impacts cell cycle progression in BC

As G2/M checkpoint was one of the differentially dysregulated pathways between ARGLU1 and pS77-ARGLU1 (Fig. 6, *A* and *B*), and MCM genes are known to be involved in the cell cycle maintenance and progression, we performed cell cycle analysis on MCF-7 cells expressing VC, WT, S77A, or S77D. Analysis showed that the percentage of cells in G2/M phase decreased significantly in WT (0.7333 ± 0.1674) when compared to the VC (6.890 ± 2.106), and substantially increased in the S77A cells (5.620 ± 0.0916) with respect to WT (Fig. 6*C*). The increase in S77A G2/M population was salvaged in the phosphomimic S77D mutants (0.5333 ± 0.0751) (Fig. 6*D*). Meanwhile, Cyclin B1 levels were assessed as a marker for the G2/M checkpoint (Fig. 6*E*). Concomitant with our flow cytometry results, Cyclin B1 expression was higher in the VC and S77A cells compared to WT and S77D. Hence, we conclude that S77 phosphorylation of ARGLU1 plays an important role in the cell cycle, especially, G2/M progression.

**Figure 6:**
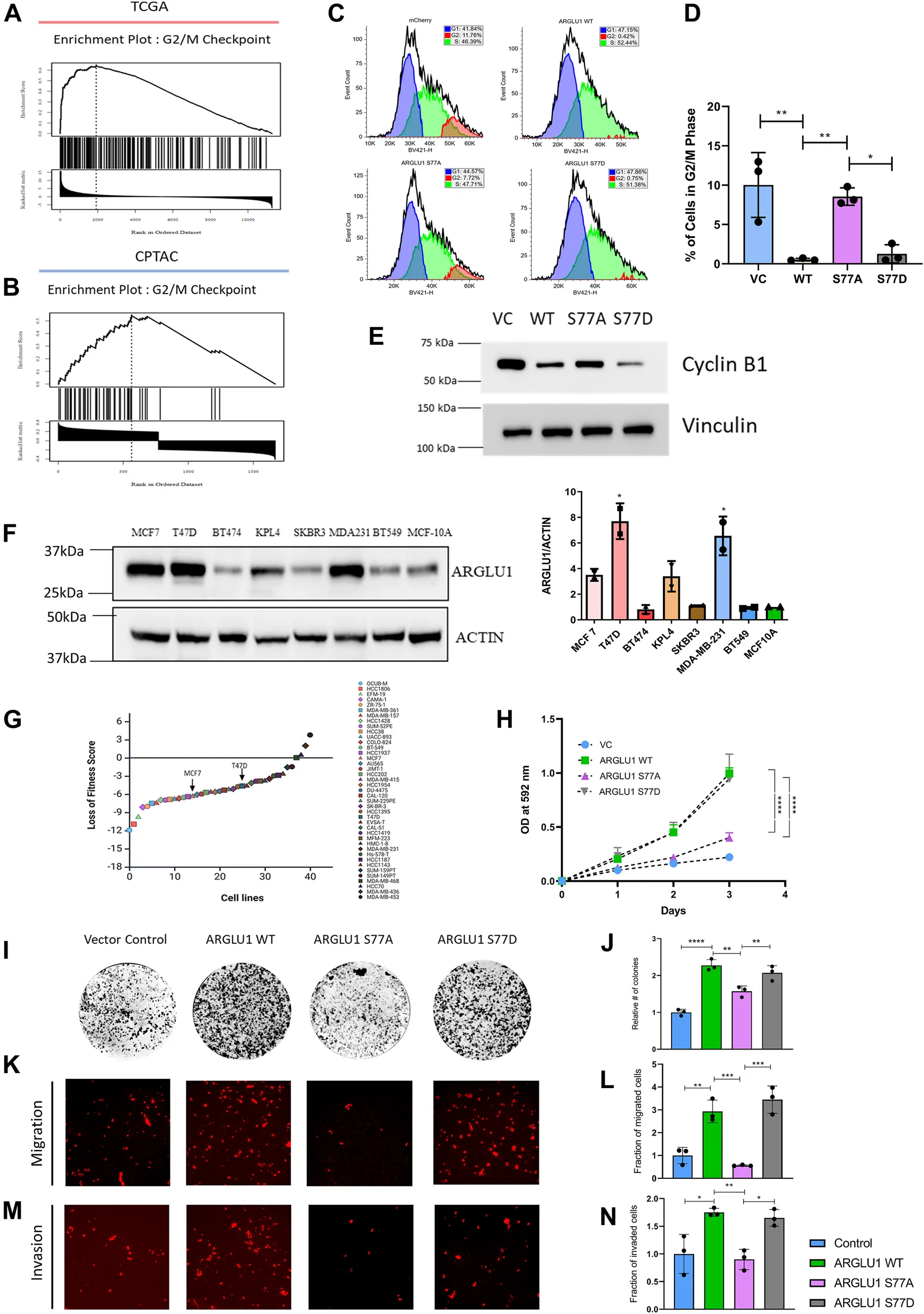
**Abrogating phosphorylation of ARGLU1 at Ser77 affects the tumorigenic potential of MCF-7 cells.***A* and *B*, enrichment plot of G2/M checkpoint between ARGLU1 and pS77-ARGLU1 in TCGA and CPTAC BRCA cohorts. *C*, representative DNA histograms of DAPI stained MCF-7 cells expressing vector control mCherry (VC), mCherry-ARGLU1 WT, mCherry-ARGLU1 S77A, and mCherrry-ARGLU1 S77D. *D*, graphical representation of percentage of cells in G2/M phase in MCF-7 cells expressing mCherry (VC), mCherry ARGLU1 WT, mCherry-ARGLU1 S77A, and mCherry-ARGLU1 S77D. Bars represent mean ± SD. Statistical analysis performed was one-way ANOVA with Tukey’s posttest for multiple comparisons. ∗*p* < 0.05 and ∗∗*p* < 0.005. *E*, Representative Western blot of Cyclin B1 in MCF-7 VC, WT, S77A, and S77D cells. *F*, *left panel*: representative Western blot demonstrating ARGLU1 levels across BC cell lines. *Right panel*: graphical representation of ARGLU1 levels across BC cell lines. MCF10A lysate was used as the baseline. Bars represent mean ± SD. Statistical analysis performed was one-way ANOVA with Tukey’s posttest for multiple comparisons. ∗*p* < 0.05. *G*, graphical representation of loss of fitness score of 41 BC cells for ARGLU1 expression loss from Project Score Database. *H*, graphical representation of the proliferation rate of MCF-7 cells stably expressing mCherry (VC), mCherry ARGLU1 WT, mCherry ARGLU1 S77A, or mCherry ARGLU1 S77D constructs by MTT assay at 0, 24, 48, and 72 h. *I*, representative colonies formed by MCF-7 cells stably expressing VC, WT, S77A, and S77D. *J*,graphical representation of relative number of colonies formed in VC, WT, S77A, and S77D. *K*, representation of migration of MCF7 cells stably expressing VC, WT, S77A, and S77D. *L*, graphical representation of relative fraction of migrated cells in VC, WT, S77A, and S77D. *M*, representation of invasion of MCF7 cells stably expressing VC, WT, S77A, and S77D. *N*, Graphical representation of relative fraction of invaded cells in VC, WT, S77A, and S77D. (*I*–*N*) Bars represent mean ± SD (n = 3). Statistical analysis performed was one-way ANOVA with Tukey’s posttest for multiple comparisons. ∗*p* < 0.05, ∗∗*p* < 0.01, ∗∗∗*p* < 0.001, and ∗∗∗∗*p* < 0.0001. ARGLU1, arginine and glutamate-rich protein 1; CPTAC, Clinincal Proteomic Tumor Analysis Consortium; TCGA, The Cancer Genome Atlas.

### Abrogating phosphorylation of ARGLU1 at Ser77 affects the tumor promoting capabilities of ARGLU1

To assess protein expression of ARGLU1 across BC cell lines, we performed Western blot on the same with MCF-10A lysate as control (Fig. 6*F*). Project Score Database showed that 37 out of 41 BCs cells had negative loss of fitness scores for ARGLU1. Among them, MCF-7 had a modest score of −6.36, indicating a strong reduction in viability and rate of proliferation upon knocking out *ARGLU1* (Fig. 6*G*). Therefore, to explore the functional effects of the phosphorylation of ARGLU1 at Ser77 on the tumorigenic potential of MCF-7 cells, VC, WT, S77A, and S77D constructs were stably transfected into MCF-7. To comprehensively assess the effects of pS77-ARGLU1 on cancer cell growth, we employed complementary short-term metabolic activity by MTT and long-term survival by clonogenic assays. Abolishing the S77 phosphorylation of ARGLU1 markedly attenuated cancer cell growth, as evidenced by a substantially diminished viability (Fig. 6*H*) and about 32% reduction in colony forming capacity in S77A cells compared to WT cells (Fig. 6, *I* and *J*). Importantly, the phosphomimic, S77D mutant completely restored the reduced proliferative capacity observed in S77A cells. Notably, the diminished colony formation emphasizes a sustained impairment of proliferative fitness in these cells. The rescue supports a phosphorylation-dependent mechanism governing the growth-promoting function of ARGLU1 in cancer cells.

To assess changes in metastatic phenotypes due to ARGLU1 phosphorylation, we utilized transwell assays with or without Matrigel, to measure both migration and invasion capacities. ARGLU1 WT cells showed about 3-fold higher migratory and 1.7-fold higher invasion capacity than VC. Both these parameters were markedly reduced in the S77A (5- and 2-folds in migration and invasion respectively) when compared to the WT. Similarly, S77D mutation rescued the phenotype with almost 6- and 2-fold increase in the proportion of migrated and invaded cells than in S77A (Fig. 6, *K*–*N*).

To evaluate the role of ARGLU1 S77 phosphorylation on three-dimensional growth capacity of the cell populations, we assessed their spheroid-forming ability under defined culture conditions. The morphometric parameters derived from perimeter, circularity, solidity, optical density, extracted from brightfield images were normalized to VC. It was observed that VC cells formed small, rounded spheroids with well-defined borders. In contrast, WT cells generated larger, elongated, and highly compact spheroids, reflected by an increased composite spheroid-forming index relative to VC (Fig. 7, *A* and *B*). The S77A cells failed to form organized spheroids and instead produced loosely packed cellular aggregates lacking clear structural boundaries with visible gaps, resulting in a significantly lower composite spheroid-forming index compared to WT. The S77D cells formed spheroids with almost 2-fold higher index when compared to S77A. We then harvested these spheroids and stained them for PCNA, E-Cadherin (E-Cad), and Vimentin (Vim). We observed that the PCNA levels were higher in the WT compared to VC and S77A, and S77D showed significantly increased staining of PCNA with respect to S77A (Fig. 7, *C* and *D*). The disorganized morphology observed in S77A spheroids suggests impaired cell–cell cohesion and architectural stability. This was confirmed by the lack of significant difference in the levels of EMT markers, E-Cad and Vim by immunohistochemistry (Fig. S2, A and B). Comparable expression levels of E-Cad and Vim across WT, S77A, and S77D spheroids exclude differential invasive potential as a cause of the poorly defined boundaries observed in S77A spheroids. This was in line with our observation that Ki67 and PCNA levels positively correlated with the pS77-ARGLU1 levels in BC patients from CPTAC cohort (Fig. 7, *E* and *F*). Our *in-vitro* data supported the tumorigenic role of ARGLU1 phosphorylation at Ser 77 as seen from the multi-omics analyses. Taken together, we conclude that disruption of ARGLU1 phosphorylation at S77 alleviated the oncogenic potential of MCF-7 cells.

**Figure 7:**
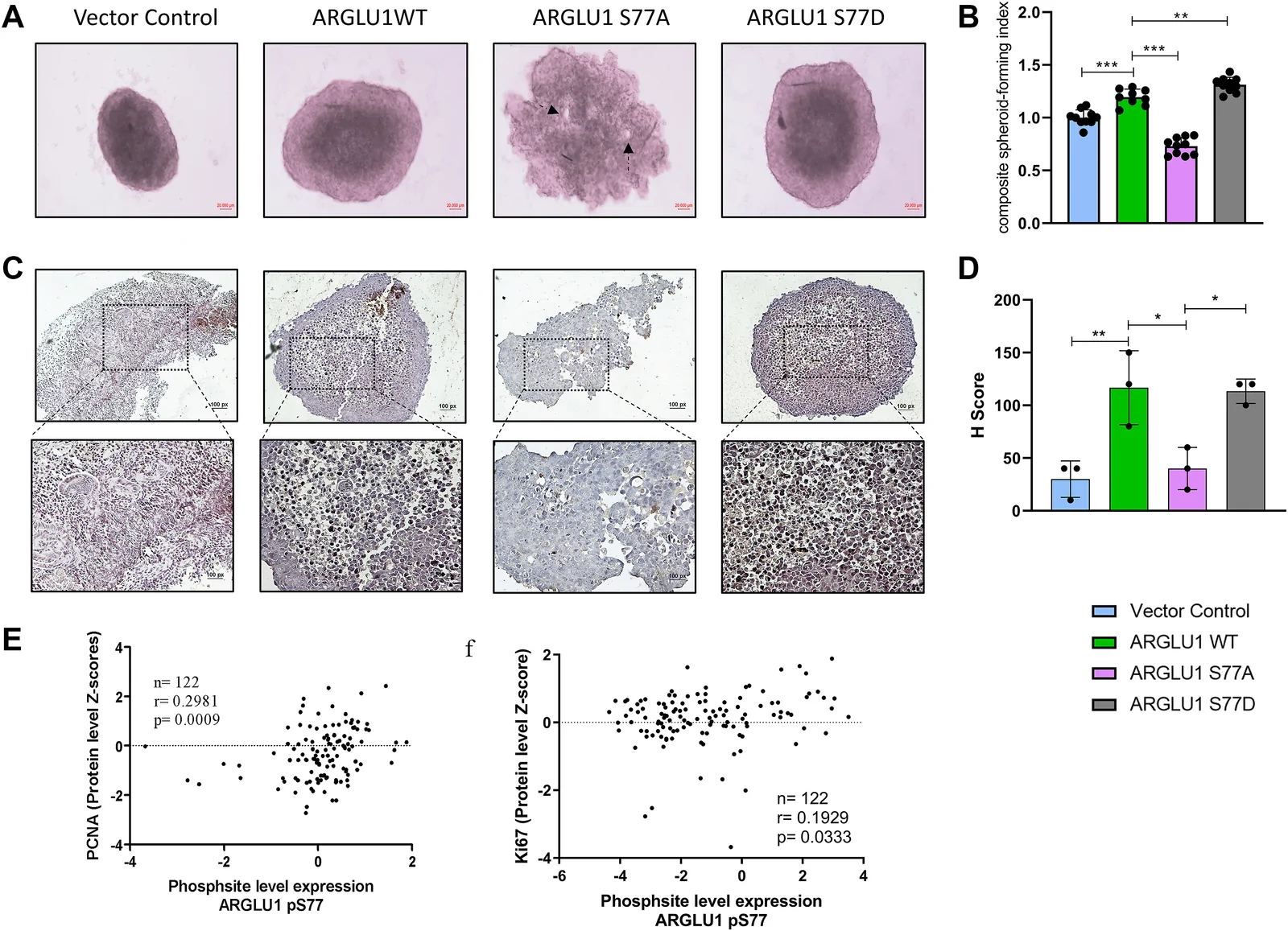
**Abrogating phosphorylation of ARGLU1 at Ser77 affects the spheroid-forming capacity of MCF-7 cells.***A*, representative image of spheroids formed by MCF-7 cells stably expressing VC, WT, S77A, and S77D. *Black arrows* represent the gaps between the cellular aggregates. *B*, graphical representation of composite spheroid forming index of VC, WT, S77A, and S77D cells. Bars represent mean ± SD (n = 10). Statistical analysis performed was one-way ANOVA with Tukey’s posttest for multiple comparisons. ∗∗*p* < 0.01 and ∗∗∗*p* < 0.001. *C*, representative images of spheroid sections stained for PCNA counterstained with Hematoxylin. *D*, Graphical representation of PCNA staining intensity as H-score. Bars represent mean ± SD (n = 3). Statistical analysis performed was one-way ANOVA with Tukey’s posttest for multiple comparisons. ∗*p* < 0.05 and ∗∗*p* < 0.01. *E*, correlation plot showing positive association between pS77-ARGLU1 and PCNA in CPTAC BRCA cohort. *F*, correlation plot showing positive association between pS77-ARGLU1 and Ki67 in CPTAC BRCA cohort. ARGLU1, arginine and glutamate-rich protein 1; CPTAC, Clinincal Proteomic Tumor Analysis Consortium; PCNA, proliferating cell nuclear antigen.

### Clinical significance of PAK1 phospho-ARGLU1 S77 axis

Results so far revealed the mechanistic and functional significance of ARGLU1 phosphorylation at Ser77. To experimentally confirm that ARGLU1 was overexpressed in breast tumor samples, we performed immunohistochemical analysis (IHC) on the high-density BC tissue microarray (TMA), in which we observed increased levels of ARGLU1 in tumor samples when compared to normal breast tissues with statistical relevance (*p* < 0.05, Fig. 8, *A* and *B*). We also endeavored to find out the differential protein levels of ARGLU1 in BC patient samples from CPTAC - BRCA dataset. It was found that ARGLU1 levels were significantly higher in tumor when compared to adjacent normal tissues (Fig. 8*C*). Similarly, PAK1 was overexpressed in tumors when compared to normal in BC TMA (Fig. 8, *D* and *E*). It was also found that PAK1 levels were significantly higher in tumor when compared to adjacent normal tissues in CPTAC cohort (Fig. 8*F*). Apart from BC, it was noted that ARGLU1 levels were higher in multiple cancers including colon cancer, lung adenocarcinoma, HNSCC and hepatocellular carcinoma (Fig. S4*A*).

**Figure 8:**
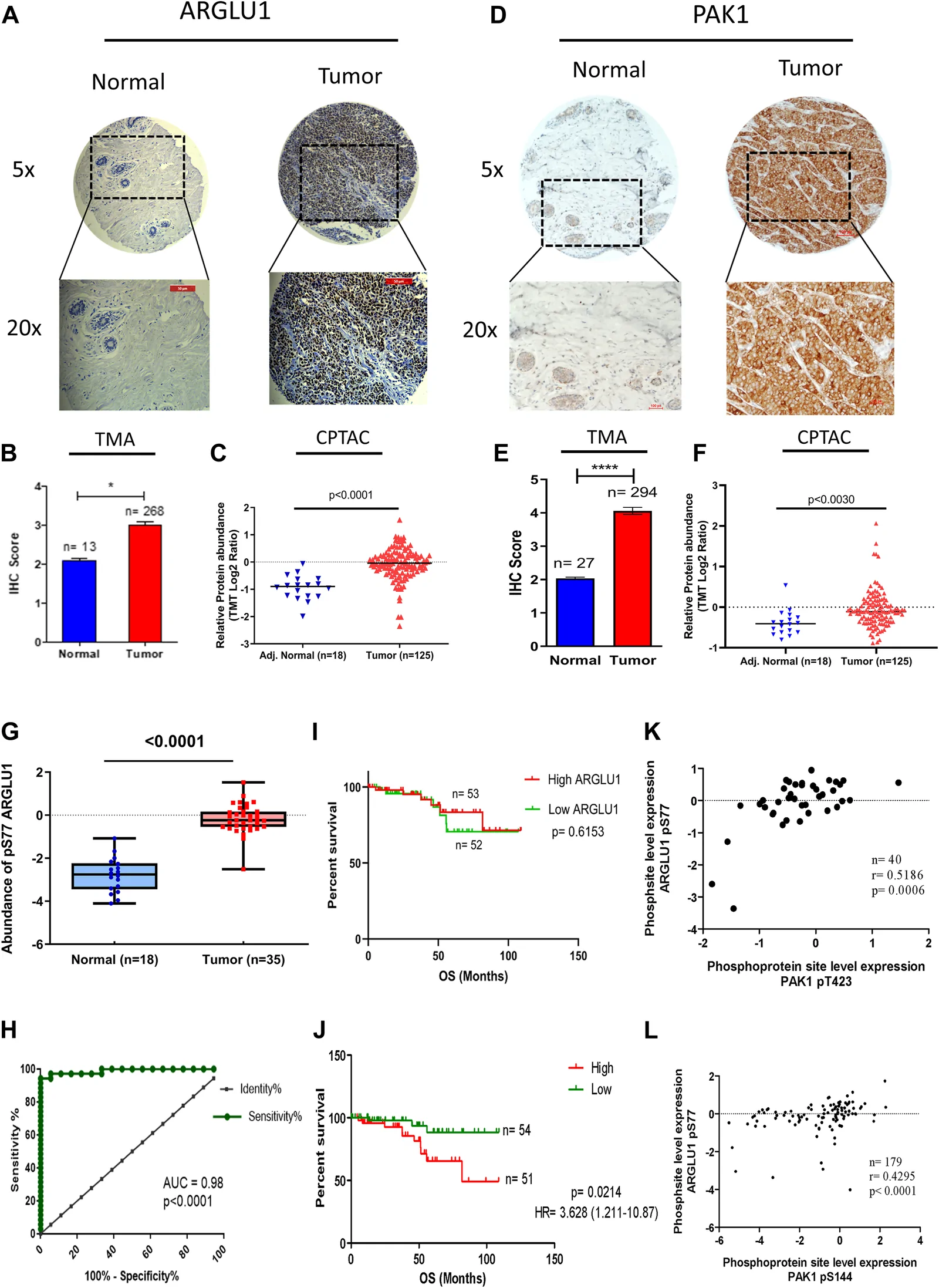
**Clinical significance of ARGLU1 and p S77-ARGLU1 in BC.***A*, representative IHC images stained for ARGLU1 in high density TMA containing breast normal and tumor tissue cores. All images were taken at 5× and 20× magnification. The scale bar represents 50 μM (the normal panel figure is derived from the same source image as the forth normal panel of Fig. S3) . *B*, graphical representation of ARGLU1 IHC scores, expressed as mean ± SEM. Mann Whitney U test was performed to assess the statistical significance. *p* ≤ 0.05 was considered significant. *C*, graphical representation of ARGLU1 levels in breast tumors as compared to adjacent normal tissues in CPTAC dataset. Statistical analysis performed was Mann Whitney U Test. *p* ≤ 0.05 was considered significant. *D*, representative IHC images stained for PAK1 in high density TMA containing breast normal and tumor tissue cores. All images were taken at 5× and 20× magnification. *E*, graphical representation of PAK1 IHC scores, expressed as mean ± SEM. Mann Whitney U test was performed to assess the statistical significance. *p* ≤ 0.05 was considered significant. *F*, graphical representation of PAK1 levels in breast tumors as compared to adjacent normal tissues in CPTAC dataset. Statistical analysis performed was Mann Whitney U Test. *p* ≤ 0.05 was considered significant. *G*, graphical representation of pS77-ARGLU1 levels in breast tumors as compared to adjacent normal tissues in CPTAC dataset accessed through cProSite. Statistical analysis performed was Mann Whitney U Test. *p* ≤ 0.05 was considered significant. *H*, Receiver Operating curve analysis of pS77-ARGLU1 in tumor *versus* normal. *I*, Kaplan–Meier plot for overall survival for low (*green*) *versus* high total ARGLU1 levels (*red*). *p* ≤ 0.05 was considered statistically significant. Graphs were generated using GraphPad Prism v9. *J*, Kaplan–Meier plots for overall survival for low (*green*) *versus* high pARGLU1-S77 levels (*red*). *p* ≤ 0.05 was considered statistically significant. Graphs were generated using GraphPad Prism v9. *K*, correlation plots between phospho-ARGLU1 levels and pPAK1 T423 and *L*, pPAK1 S144 levels in patient samples of TCGA-BRCA dataset. Spearman coefficient was used to quantify the correlation. *p* ≤ 0.05 was considered statistically significant. ARGLU1, arginine and glutamate-rich protein 1; CPTAC, Clinincal Proteomic Tumor Analysis Consortium; IHC, immunohistochemical analysis; PAK1, p21-activated kinase 1; TMA, tissue microarray.

We next proceeded to understand the clinical significance of phosphorylation of ARGLU1 at Ser77. Analyzing the CPTAC data from cProSite showed that pS77-ARGLU1 levels were higher in BC when compared to normal tissues (Fig. 8*G*). ROC analysis of the data obtained an AUC of 0.98, indicating superior discriminatory power of pS77-ARGLU1 to distinguish tumor and normal. These results indicated that this phosphorylation is a highly tumor enriched event and exhibited the diagnostic potential of pS77-ARGLU1 in BC (Fig. 8*H*). Like the total ARGLU1, pS77-ARGLU1 levels also were found to be higher in other cancers such as colon cancer, ovarian cancer, and HNSCC (Fig. S4*B*). Furthermore, in BC pS77-ARGLU1 levels were also strongly associated with advanced stages of the disease (Fig. S4*C*). Intriguingly, stemness score positively correlated with phospho-ARGLU1 S77, indicating that this phosphorylation event might be promoting stem cell-like properties in BC (Fig. S4*D*).

It is noteworthy that total ARGLU1 levels did not show significant impact on the survival outcomes of BC patients (Fig. 7*I*). Most importantly, BC patients with higher pS77-ARGLU1 had about 3.5-times (95% CI 1.2–10.8) poorer overall survival rate when compared to those with lower pS77-ARGLU1 levels (*p* = 0.02) (Fig. 7*J*). Furthermore, the levels of pS77-ARGLU1 positively correlated with that of phosphorylated PAK1 (sites essential for its catalytic activity) at T423 and S144 in BC from TCGA cohort (Fig. 7, *K* and *L*). Taken together, our data implies the advanced clinical relevance of the ARGLU1 phosphorylation event and the significance of PAK1-ARGLU1 axis in BC.

## Discussion

ARGLU1 is a poorly studied protein and its relevance in cancers is immensely underexplored. (15). An elaborate study by Li *et al.* showed that ARGLU1 mRNA levels were significantly reduced in gastric cancer and that lower expression of ARGLU1 correlated with advanced TNM stage of gastric cancer. *In vitro*, knocking down of *ARGLU1* in gastric cancer cell lines such as HGC27 and MKN45 increased the clonogenicity and proliferation rate of these cells and promoted tumor growth *in vivo*. Moreover, they also found that ARGLU1 enhanced the transcription of DNA repair genes such as MLH3, MSH2, MSH3, and MSH6 (17). Similar results were observed in HNSCC, where knocking down of *ARGLU1* increased the tumorigenic potential of HNSCC cells both *in-vitro* and *in vivo* (19). In contrast, Zhang *et al.* showed that knocking down *ARGLU1* affected the colony forming ability and anchorage independent growth of MCF-7 cells (15). This agreed with more recent study by Peter Pelka’s group. They reported that overexpressing ARGLU1 in the fibrosarcoma cell line – HT1080 improved the proliferation rate of the cells, while silencing ARGLU1 inhibited the growth. Besides, overexpression of ARGLU1 increased the resistance to genotoxic drugs by promoting DNA repair through promoter-proximal pausing of RNA Pol II (18). Thus, the role of ARGLU1 in tumorigenesis appears to be highly context-dependent. Interestingly, existing knowledge of posttranslational modification of ARGLU1 is limited to ubiquitination by DLT leading to its degradation, thereby promoting HNSCC. According to Phosphosite database, phosphorylation at S77 of ARGLU1 is frequently reported in multiple high-throughput studies (Fig. S5). Yet, at the molecular level, the upstream kinase which could possibly phosphorylate ARGLU1 at Ser77 has not been reported as far as we know. To the best of our knowledge, our study is the first to explore the mechanistic and clinical significance of phosphorylation of ARGLU1 at Ser77.

By KISS assay, we found ARGLU1 as a potential substrate of PAK1 getting phosphorylated at Ser 77. Further experimental validations such as coimmunoprecipitation, *in-vitro* kinase assay and PhosTag gel showed that ARGLU1, indeed, was a *bona fide* substrate of PAK1. PAK1 activity was found to be essential for regulating the subcellular localization of ARGLU1. Being an oncogenic protein kinase, PAK1 phosphorylates a myriad of substrates, and hence its inhibition/knockdown causes an overall change in the phosphoproteomic landscape of the cell. Therefore, PAK1 may influence the localization of ARGLU1 *via* multiple axes. Our multi-omics analysis on BC patient samples indicated possible role of pS77-ARGLU1 in upregulation of E2F and Myc targets and genes involved in G2/M checkpoint.

Therefore, we embarked to study the mechanistic significance of pS77-ARGLU1 in altering cMyc-regulated transcriptional progress. We first confirmed the colocalization and interaction of cMyc with ARGLU1 in MCF-7 cells. cMyc is known to bind to E-box elements on gene promoters thereby bringing about the transcription. We designed promoter luciferase experiments to delineate the role of PAK1 and ARGLU1, significance of pS77-ARGLU1, and PAK1-ARGLU1 signaling axis in cMyc-mediated E-box activity. These data indicate that phosphorylation of ARGLU1 positively regulate cMyc responsive transcription. However, further studies are required to understand the precise molecular events underpinning the altered cMyc activity through phosphorylation of ARGLU1. We also estimated the MCM gene expression levels using qRT-PCR, as representative of genes with E-box promoter. As expected, compared to VC, ARGLU1 WT cells showed higher MCM transcript levels, with S77A falling back to levels comparable to VC, and S77D showed expression similar to WT.

Consistent with these findings, cells expressing ARGLU1 WT showed significantly higher rate of progression through cell cycle, when compared to both VC and S77A. The trend was reiterated by cyclin B1 expression across these groups. Concordant with the earlier findings (15), overexpressing ARGLU1 increased the proliferation rate and clonogenicity of MCF-7 cells. On the other hand, the cells stably expressing ARGLU1-S77A proliferated at a slower rate and formed lower number of colonies. Similarly, attenuation of transwell migration and invasion potential was observed in cells expressing phospho-deficient mutant. Also, the spheroids formed by the cells expressing the phospho-deficient mutant was less solid and compact than those formed by the cells expressing ARGLU1 WT. In contrast to WT spheroids, S77A spheroids exhibited poorly defined boundaries and a loosely organized cellular architecture, characterized by evident intercellular gaps. This altered morphology suggests impaired cell–cell adhesion and reduced structural cohesion. The loosely formed aggregates indicate the differential proliferation rate of the spheroids as substantiated by the differential expression levels of PCNA. This agrees with the earlier report that mRNA levels of PCNA is elevated in cells overexpressing ARGLU1 (18). The lack of compaction observed in the S77A spheroids was not due to superior migratory potential as ascertained by similar E-Cadherin and Vimentin expression across the groups. Results from the publicly available datasets demonstrated that pS77-ARGLU1 levels positively correlated with the stemness score, Ki67 and PCNA levels in BC, corroborating our experimental data. Besides, pS77-ARGLU1 levels were highly elevated in BC, with a strong discriminatory power of AUC = 0.98, and was associated with advanced clinical stage of the disease and poor overall survival. En masse, it signifies the immense clinical relevance of this phosphorylation event.

To the best of our knowledge, this is the first study to explore the pS77-ARGLU1, its upstream kinase and downstream mechanistic and clinical significance. Nevertheless, we believe that this PAK1 driven phosphorylation of ARGLU1 at Ser 77 could serve as an excellent biomarker and a therapeutic target, upon validation in larger sample size, across multiple cohorts.

## Experimental procedures

### Cell lines and reagents

MDA MB-231, MCF-7, T47D and HEK293T cell lines were purchased from American tType Culture Collection. T47D cells were grown in RPMI – 1640 supplemented with 10% fetal bovine serum (FBS) and Insulin. Other cells were grown in Dulbecco's modified Eagle's medium (DMEM) high glucose supplemented with 10% FBS. The following primary antibodies were used for Immunoprecipitation and Western blot analysis: anti-PAK1 (CST #2602), anti-ARGLU1 (Invitrogen #PA5-66041), anti-PARP (CST #9532), anti-alpha Tubulin (Abcam #Ab28439), anti-mCherry (Abcam #ERP20579), HRP-conjugated anti-rabbit (Rockland, #611-1302), and anti-mouse (Rockland #610-1302). The following antibodies were used for Immunofluorescence analysis: anti-PAK1- AF 488, ARGLU1 (Novus Biologicals #NBP1-87921), and anti-rabbit Alexa Flour 546 (Invitrogen #A11012). For IHC, the following antibodies were used: anti-ARGLU1(Novus Biologicals #NBP1-87921), anti-Ki67(PathnSitu #PM210), anti-PCNA (PathnSitu #PR065). mCherry-ARGLU1 and FLG--ARGLU1 plasmids were kind gifts from Dr Carolyn L. Cummins (Department of Pharmaceutical Sciences, University of Toronto). pcDNA3.1 HA-Myc construct was a kind gift from Prof. Mahalingam, Department of Biotechnology, IIT Madras. pCMV6M-PAK1-T423E (#12208), pCMV6M-Pak1K299R (#12210), and pGL4.23-5xE-box (##206841) plasmids were purchased from Addgene. PAK1 siRNA was procured from CST (CST #6536). PAK1 inhibitor IPA3 was purchased from Tocris (#3622). Site Directed mutagenesis was carried out using The QuikChange II site–directed mutagenesis kit (Agilent #200524) as per the manufacturer’s protocol. The primers used were FP:5′-tcgggcggggccgaggcgcgc-3′ and RP:5′-gcgcgcctcggccccgcccga-3′ for Ser 77 to Ala mutant and FP 5′agcagcaagcacaacaagaagcgc 3′ and RP 5′tcgaactccgctttcttctc 3′ for Ser77 to Asp.

### Transfections

siRNA transfection was carried out using RNAimax (Invitrogen #13778100) as per the manufacturer’s protocol. Briefly, 55 pMol of siRNA was transfected into cells in 60 mm dishes at 60 to 70% confluency with OptiMEM (Thermo Fisher Scientific 22600050) medium. A total of 1 ml of complete medium was added 6 h posttransfection and was changed to complete media 24 h posttransfection. After 48 h after transfection, the cells were harvested.

Plasmid DNAs were transfected using Fugene HD (Promega #E2311) as per the prescribed protocol. Shortly, when cells were around 50% confluent, plasmids were transfected in OptiMEM media (Plasmid: Fugene – 1:3). 24 h posttransfection, cells were changed to complete media and harvested after 48 h.

### KISS

KISS was performed as described previously (21, 22) modifications. Briefly, MDA MB 231 PAK1 KOt cells were lysed with radioimmunoprecipitation assay (RIPA) buffer supplemented with protease inhibitor cocktail (Roche, #04693116001) and PhoStop (Roche, # 4906845001). 2 mg of the protein lysate was incubated with 400 pmol of GST-PAK1 for 1 h at 4 °C with rotation. Later, 40 μl of GST beads were added to the lysate and incubated at 4 °C overnight. The beads were then washed at least thrice with TED buffer (20 mM Tris HCl pH 7.5, 1 mM EDTA, and 1 mM DTT). *In vitro* kinase assay was set up with or without ATP (25 μM). The samples were then denatured by adding guanidine hydrochloride and centrifuged at 10,000×*g* for 5 min. The resulting supernatant was reduced using 0.1 M DTT and alkylated using 0.2 M iodoacetamide. The protein was precipitated using ice cold methanol and chloroform and dried using speed vac. The samples were then reconstituted using 6 M urea and 50 mM ammonium bicarbonate. The proteins were digested overnight at 37 °C using Trypsin (Promega #V5280). After digestion, the phosphor-peptides were enriched using Titansphere Phos-TiO kit (GL sciences) as per the manufacturer’s protocol and then desalted using C-18 Zip tips (Millipore, #ZTC18S). The samples were dried and store in −20 °C till further use.

### LC-MS/MS

The desalted peptides were separated using Dionex UltiMate 3000 RSLC nano System (Thermo Fisher Scientific) associated with an Orbitrap Elite Mass Spectrometer (Thermo Fisher Scientific). A step gradient of 1 to 99% solvent B (0.1% formic acid [FA] in 80% acetonitrile) at a flow rate of 300 nl/min over 100 min was used to separate the peptides. A survey full scan MS (from *m/z* 375–1200) was acquired in time-dependent acquisition mode with a resolution of 120,000. Peptides with charges 2 to 6 were considered for MS acquisition and were then selected in a data-dependent manner for MS2 scan with Quadrupole filter. MS/MS spectra were acquired using electron transfer dissociation. Precursor ions were isolated in the quadrupole mass filter with an isolation window of 2.0 m/z and fragmented *via* electron transfer dissociation. MS2 spectra were acquired in the Orbitrap at a resolution of 30,000.

### Mass spectrometry data analysis

The mass spectrometry-derived data was searched against the human proteome from UniProtKB using Comet algorithm 2024.01 rev. 1 (23), through SearchGUI version [4.3.10] (24). Search parameters including trypsin digestion, maximum of two missed cleavage, carbamidomethylation at cysteine was set as fixed modification, while Phosphorylation of S (+79.966331 Da), Phosphorylation of T (+79.966331 Da), and oxidation of methionine as variable modification. Precursor and fragment ion mass tolerance was set to 10 ppm each. Peptides and proteins were inferred from the spectrum identification results using PeptideShaker version 3.0.9 (25). Peptide spectrum matches, peptides and proteins were validated at a 1.0% false discovery rate estimated using the decoy hit distribution. Posttranslational modification localizations were scored using the phosphoRS score [PMID 22073976] with a threshold of 95.0 as implemented in the compomics-utilities package [PMID 21385435]. A phosphoRS score above 0.75 was considered as a confident localization.

### IHC

All work was carried out complying with the Declaration of Helsinki. High-density BC TMA was procured from US Biomax (#BR6161). First, the sections were deparaffinized using xylene and, rehydrated with a gradient of 100-50% alcohol. Following, endogenous peroxidase blocking with H_2_O_2_ and antigen retrieval was carried out using heated TRIS-EDTA (pH 9.2). The slides were incubated with primary antibody overnight at 4 °C. The slides were washed, incubated with HRP-secondary antibody for 1 h, followed by the addition of 3,3′,5,5′-Tetramethylbenzidine substrate. The sections were then counterstained with hematoxylin and mounted using dibutyl phthalate polystyrene xylene mountant for imaging and scoring purposes. The slides were visualized using the Leica DM 2000 LED light microscope and Leica Application Suite was used to capture the images at different magnifications. The percentage of cells stained positive for the marker was scored 0 to 4 for 0%, 1 to 25%, 26 to 50%, 51 to 75%, and > 75% respectively. The intensity was given as 1 +, 2 +, and 3 + for low, medium, and high. Finally, to obtain final IHC score, the % score and the intensity were added (Range = 0–7).

### Western blot

Cells were lysed using RIPA buffer supplemented with protease inhibitor cocktail (Roche, #04693116001) and phosphatase inhibitor (Roche, 4906845001). The total amount of protein in the lysates was estimated using Bio-Rad DC protein estimation kit (#5000111) as per the manufacturer’s protocols. The samples were denatured by boiling with 6 X SDS PAGE loading buffer at 95 °C. The denatured samples were resolved in SDS-PAGE followed by transferring onto a nitrocellulose membrane. The membranes were then blocked with 5% non-fat dry milk, followed by incubation with the indicated antibodies at recommended dilutions at 4 °C overnight. After appropriate number of washes with PBS, the blots were incubated with rabbit or mouse HRP-conjugated secondary antibody for 1 h at RT. The protein bands were visualized by using Enhanced Chemiluminescent kit (Bio-Rad #1705061) in Chemi Doc Imaging system (Bio-Rad). The intensity of the bands was measured using Image Lab software.

### Coimmunoprecipitation

The indicated cells were cotransfected with respective plasmids. 48 h post transfection cells were lysed using NP40 lysis buffer. After estimating the amount of protein, the lysates were precleared with A/G beads (Santa Cruz #SC-2003). The lysates were then incubated with the mCherry antibody at 4 °C overnight with rotation. The next day, the lysates were incubated with 40 μl of A/G beads for 4 h at 4 °C. The beads were then washed with ice-cold PBS + 0.1% Triton X-100 thrice. The samples were then eluted by adding 6 X SDS loading buffer, vortexed briefly, and boiled at 95 °C for 5 min. The samples were then centrifuged at 14,000 rpm for 10 min. The supernatant was loaded in 6% SDS-PAGE. 5% of the lysate was used as input. Similarly, T47D and T47D PAK1 KO cell lysates were processed for endogenous interaction by immunoprecipitating with anti-PAK1 antibody overnight at 4 °C.

### *In vitro* kinase assay

*In vitro* kinase assay was set up with GST-PAK1 and GST-ARGLU1 (63–191 amino acids; NovoProlabs, cat #525497) using ϒp^32-^ ATP as described previously. Similar reactions were set up for myelin basic protein (positive control) and GST (negative control). After incubating the reaction mixtures at 30 °C for 30 min, the reactions were stopped by adding 6 X SDS sample loading buffer. The samples were then resolved in 12% SDS-PAGE and transferred to a nitrocellulose membrane. After visualizing the proteins using Ponceau stain, the blots were exposed to Phosphor screen overnight. The radiolabeled bands were then visualized using Typhoon FLA 9500 phospho-imaging system.

### PhosTag gel analysis

T47D cells were transfected with PAK1 siRNA or PAK1T423E. After 48 h of transfection, cells were lysed in EDTA-free RIPA buffer. The samples were then denatured and resolved in 8% SDS-PAGE containing 5 μM of PhosTag (Wako Chemicals Cat. No: 304-93526.) and 10 μM MnCl_2_ as per the manufacturer’s instruction and blotted on a nitrocellulose membrane for further analysis. The cell lysates were treated with Lambda Phosphatase (NEB, P0753S) following the manufacturer protocol was used as control.

### Immunofluorescence microscopy

MCF-7 cells (50,000 cells/well) were seeded onto coverslips placed in six well plates. After 24 h of seeding, the cells were fixed with 4% paraformaldehyde (PFA) for10 min at 4 °C and 10 min at RT. The cells were then treated with antibody permeabilization buffer (0.1% Triton X-100 in PBS) for 10 min, followed by blocking with 2% BSA solution. The cells were then incubated with the recommended dilutions of the indicated primary antibodies overnight at 4 °C. After appropriate washes, the cells were incubated with the secondary antibody, stained with 4′,6-diamidino-2-phenylindole (DAPI) and mounted to glass slides using VectaShield (Vecta Laboratories #H1000). The stained cells were imaged using Olympus Flouview 3000 confocal microscopy. For colocalization analysis, Mander’s colocalization coefficients were calculated by applying Costes auto-threshold using JACoP tool from BioP plugin in the ImageJ software.

### Nuclear-cytoplasmic extraction

48 h posttransfection, cells were trypsinized and pelleted by centrifugation at 1080 rpm for 5 min. After washing the pellets with ice cold PBS, the cell pellets were incubated with the cytosolic extraction buffer (10 mM Hepes, 10 mM KCL, 100 μM EDTA, 100 μM EGTA, 1 mM DTT, 0.075% NP-40, and PIC) for 30 min on ice with gentle agitation at regular intervals. The samples were then centrifuge at 12,000 rpm for 5 min and the resultant supernatant was collected as the cytosolic extract while the pellet contained the nuclear extract. The pellets were then washed with cytosolic extraction buffer thrice with centrifugation at 12,000 rpm for 5 min each time. After this, the pellets were incubated with nuclear lysis buffer (20 mM Hepes, 400 mM NaCl, 1 mM EGTA, and PIC) for 1 h on ice with intermittent vertexing every 10 min. The samples were then sonicated for 5 s ON/10 s OFF for three cycles, followed by centrifugation at 12,000 rom for 5 min. The supernatant was collected as the nuclear extract and stored at −80 °C until further use.

### *In vivo* metabolic labeling

Cells were seeded in 60 mm dishes and transfected with mCherry-ARGLU1 WT and mCherry-ARGLU1 S77A plasmids. After 48 h, the cells were labeled with P^32^-orthophosphoric acid in phosphate-free DMEM media and incubated overnight. The lysates were then collected and immunoprecipitated with anti-mCherry and subjected to Western blot. The samples were then resolved in 8% SDS-PAGE and transferred to polyvinylidene fluoride membrane. After visualizing the proteins using Ponceau stain, the blots were exposed to Phosphor screen overnight. The radiolabeled bands were then visualized using Typhoon FLA 9500 phospho-imaging system.

### MALDI-ToF MS of ARGLU1 peptides

We designed peptides flanking the Serine 77- DRERASSPPDRIDIFGRTVS (WT) and phospho-deficient - DRERASAPPDRIDIFGRTVS (S77A). We commercially procured the custom purified peptides and set up an *in-vitro* kinase assay with or without GST-PAK1. The peptides were then extracted with 50% v/v acetonitrile in 0.1% v/v trifluoroacetic acid and proceeded for matrix-assisted laser desorption ionization mass spectrometry. The mass spectrometry analysis was performed using MALDI-ToF (UltrFleXtreme, Bruker Daltonics). To acquire MS spectrum, 1 μl of the elute was mixed with 1 μl a-cyano-4-hydroxycinnamic acid matrix (50% acetonitrile in 0.1% TFA water) and 1 μl of this mixture was spotted on to a MALDI target plate. Final mass spectra were obtained by averaging 2500 laser shots collected at five different positions.

### Cell cycle analysis

After 48 h of transfection, the cells were trypsinized and fixed with 4% PFA. Fixed cells were washed with PBS and then stained with 10 μg/ml DAPI, 1 μg/ml RNase A in PBST with 0.1% (v/v) Triton X-100 for 30 min at RT in dark. DAPI was read at Ex/Em 405-450/50 in a linear mode, 20,000 events per sample were acquired at low flow rate using BD FACS symphony. The cell-cycle profiles were set down with untransfected control and in the mCherry positive cells transfected with the vector control. Cells positive for mCherry (Ex 552/Em610/20) were gated onto DAPI to profile the different phases of cell cycle in VC, WT, S77A, and S77D transfected cells.

### Generation of stable ARGLU1 overexpression cell lines

Plasmids expressing mCherry, mCherry-ARGLU1 WT, mCherry-ARGLU1 S77A, and mCherry ARGLU1 S77D were transfected into MCF-7 cells using Takara Xfect transfection reagent as per the manufacturer’s protocol. After 48 h of transfection, cells were selected using puromycin (1 μg/ml) for 21 days. After selection, isolated colonies were collected and screened for positive clones by mCherry Western blot.

### Luciferase assay

The indicated cells were cotransfected with E-box promoter luciferase construct, β- Galactosidase plasmid and indicated plasmids. After 48 h of transfection, the cells were lysed and assayed using luciferase assay system kit (Promega, E4030) as per manufacturer’s protocol. The RLUs were normalized with β- Galactosidase readings.

### qRT-PCR

Total RNA collected from the MCF-7 cells stably expressing mCherry, mCherry-ARGLU1 WT, mCherry-ARGLU1 S77A, and mCherry-ARGLU1 S77D were converted to cDNA using Takara PrimeScript RT Reagent kit (RR037A) following manufacturer’s protocol. Using Applied Biosystems Quantstudio 7-Flex, MCM gene expression was quantified with the help of TB Green Premix ExTaq TM II (RR8208). The following gene-specific primers were used in the study.

MCM2 FP 5′CTATGATGGCTCCCTCAACAC3′ and RP 5′TCACATCTTCATCGGTCAGTTC3′.

MCM3 FP 5′CAAGGATCACCAGACCATCAC3′ and RP 5′TCACCAGGCTTCGCTTTATC3′.

MCM6 FP 5′ TGATTCGTCCTGAGAGAAACAC3′ and RP 5′CCGACACAGGTAAGGGTAAAC3′.

MCM7 FP 5′ CGTCAGCGTCACTGGTATTT3′ and RP 5′TCCTCACTCTTGTTCATCTTCAC3′.

### Proliferation assay

5000 cells/well were seed in 96-well plates and incubated at 37 °C and 5% CO_2_. MTT reagent was added at 24 h, 48 h or 72 h post cell seeding and incubated at 37 °C for 4 h. After incubation, the formazan crystals thus formed were solubilized using DMSO and the absorbance was measured at 592 nm.

### Clonogenic assay

A total of 5000 cells/well were seeded in a 6-well plate and incubated for 7 days in an incubator at 37 °C and 5% CO_2_. Colonies thus formed were fixed with and stained with 5% w/v Crystal violet. After removing the excess stain with PBS washes, the plates were dried and imaged in Chemi Doc MP imaging system. The colonies were counted using Image J.

### Trans well migration and invasion assays

A total of 2000 Cells/well were seeded in the upper chamber of Corning co-star 24-well inserts plate. The upper chamber contained serum free DMEM media while the lower chamber contained DMEM with 10% FBS. After overnight incubation, the unmigrated cells were carefully removed using a wet cotton swab. The migrated cells were then fixed with 4% PFA and imaged in upright fluorescent microscope. Number of cells per each filed were counted using ImageJ.

For invasion assay, the upper chamber of the corning co-star 24 well inserts were coated with Matrigel (final concentration 300 μg/ml) as per the manufacturer’s protocol. A total of 3000 cells/well were seeded and incubated overnight at 37 °C and 5% CO_2_ incubator. The next day cells were fixed, imaged and counted as described previously.

### Spheroid formation assay

A total of 5000 cells/well were seeded in the ultralow attachment plates and incubated for 6 days at 37 °C and 5% CO_2_. After 6 days, the spheroids were imaged using a bright field microscope. The perimeter, area, circularity, solidity, and optical density of the spheroids were measured using ImageJ. We derived a quantitative, spheroid forming index, combining these parameters, and plotted it for different experimental groups. For IHC analysis, the spheroids were washed in PBS, fixed for 1 h in 4% PFA and embedded in 2% agarose in 4% PFA. Fixed spheroid agarose balls were stored in 10% formalin fixative solution until further use. The agarose balls were embedded with paraffin wax and 5 μM sections were cut using a Leica RM2235 microtome. The sections thus obtained were immunostained for PCNA, E-Cad, and Vim as mentioned earlier. The sections were given a score of 0, 1+, 2+, and 3+ based on the staining intensity, and % of cells stained positive was noted. H-score was calculated by combining both these values (intensity x % positivity).

### Clinical correlation from publicly available databases

The data pertaining to the abundance of ARGLU1 and pS77-ARGLU1 in BC patients of CPTAC cohort was accessed through cProSite webtool (26). The correlation data of pARGLU1 S77 levels, with, pPAK1 S144, pPAK1 T212, pPAK1 T423, Ki67, and PCNA levels and the patient survival data for pS77-ARGLU1 and ARGLU1 were obtained from cBioPortal- TCGA Firehose Legacy and Pan Cancer datasets.

### Multi-omics data analysis

LinkedOmics suite (27) was used to deduce the genes differentially correlated (RNAseq; FPKM_Normalizedlog2) with the ARGLU1 levels (Proteome; Tumor_TMT_LogRatio) or pS77- ARGLU1 levels (Phosphoproteome; Tumor_TMT_LogRatio) in BC patients of the CPTAC_BRCA dataset and TCGA_BRCA dataset. The list of genes thus obtained was subjected to Gene Set Enrichment Analysis with the Hallmark 50 gene set (28) using the Webstalt online tool (29).

### Statistical analysis

All statistical analyses were performed using PRISM 9. *p* < 0.05 was considered statistically significant for all the analyses.

## Data availability

The data supporting the findings of this study are available from the corresponding author upon reasonable request.

## Supporting information

This article contains supporting information.

## Conflict of interest

The authors declare that they have no conflicts of interest with the contents of this article.

## Supporting information

Supplementary Table S2

Supplementary Figures With Legends
